# Radiological and Clinical Outcomes after Anterior Cervical Discectomy and Fusion (ACDF) with an Innovative 3D Printed Cellular Titanium Cage Filled with Vertebral Bone Marrow

**DOI:** 10.1155/2022/6339910

**Published:** 2022-04-26

**Authors:** Frizzi Mayer, Franziska Heider, Florian Haasters, Christoph Mehren

**Affiliations:** ^1^Spine Center, Schoen Clinic Munich-Harlaching, Harlachinger Str. 51, 81547 Munich, Germany; ^2^Academic Teaching Hospital of the Ludwig-Maximilians-University (LMU) Munich, Marchioninistr. 15, 81377 Munich, Germany; ^3^Department of Orthopaedics and Trauma Surgery, Musculoskeletal University Center Munich (MUM), University Hospital LMU Munich, Marchioninistr. 15, 81377 Munich, Germany; ^4^Paracelsus Medical University (PMU) Salzburg, Strubergasse 21, 5020 Salzburg, Austria; ^5^Center of Knee, Hip, Shoulder and Elbow Surgery, Schoen Clinic Munich, Harlachinger Str. 51, 81547 Munich, Germany; ^6^Academic Teaching Hospital of the Paracelsus Medical University (PMU), Strubergasse 21, 5020 Salzburg, Austria

## Abstract

**Objectives:**

To assess the clinical and radiological outcomes after ACDF with 3D printed cellular titanium cages filled with bone marrow and to compare the clinical and radiological results with the current scientific literature.

**Methods:**

ACDF was performed monosegmentally under standardized conditions. X-rays were analyzed to determine the range of motion, fusion rates, and subsidence preoperatively and 3 and 12 months postoperatively. Clinical outcome measurements included neck disability index (NDI), visual analogue scale (VAS) for brachialgia and cervicalgia, and patient satisfaction.

**Results:**

18 patients were included in the study. The mean RoM decreased from 7.7° ± 2.6 preoperatively to 1.7° ± 1.1° after 3 months and 1.8° ± 1.2° 12 months after surgery. The fusion rates were at 94.4% after 3 and 12 months. The mean subsidence was 0.9 mm ± 0.5 mm 3 months postoperatively and 1.1 mm ± 0.5 mm 12 months after surgery. The mean NDI improved significantly from preoperatively to 12 months postoperatively (34.6 ± 6.2 and 3.4 ± 4.1, respectively). The VAS-neck also showed a large improvement from 5.8 ± 2.2 before and 1.3 ± 1.4 12 months after surgery, as did the VAS-arm (6.4 ± 1.5 and 0.9 ± 1.6, respectively). Patient satisfaction was high throughout the follow-up period.

**Conclusion:**

ACDF with a 3D printed titanium cage resulted in fast fusion without pathological subsidence. In comparison to other cage materials such as PEEK, the 3D printed titanium cage was noninferior in regard to its fusion rate and clinical results.

## 1. Introduction

There are numerous and ever-increasing treatment options for patients with radicular pain resulting from cervical disc herniation or stenosis [1]. Most of the patients can be treated conservatively by pain management (WHO analgesic ladder + analgesics for neuropathic pain + coanalgesics if indicated), physio-/ergotherapy, manual therapy, physical rest, and periradicular infiltration if indicated [1]. An operative intervention is indicated if neurological deficits occur or if the pain is not manageable despite all conservative treatment possibilities [2]. The common purpose of all different surgical techniques is the decompression of the neural elements by removing the cervical intervertebral disc, stabilization of the spinal segment(s), and restoration of intervertebral height and cervical lordosis [3–6].

In 1958, Cloward introduced the anterior approach to the cervical spine to perform a discectomy and interbody fusion with an autologous cylindrical dowel of bone from the patient's ilium to maintain disc height [3]. In the same year, Smith and Robinson developed a technique using rectangular tricortical iliac crest bone blocks [4]. Autologous bone grafts combine three distinct characteristics related to bone formation: osteoproduction, osteoinduction, and osteoconduction [7]. Various methods for achieving interbody fusion after discectomy have been described, but the rationale for the choice between the given options remains unclear [8, 9]. To overcome the problems arising from using bone graft (resorption, subsidence, and donor site morbidity), various materials have been used as substitutes [7, 10] and the first cages were implanted in the late 1990s [11–13]. Different materials (including polyetheretherketone (PEEK), carbon fiber, and titanium) as well as structures and forms have been used in the past, which all show relevant advantages and disadvantages [9, 10, 13]. Lately, PEEK cages have become standard in many countries because of mainly two advantages: (1) they are cost-efficient and (2) its radiolucency facilitates the postoperative radiographic fusion control [14]. On the other hand, PEEK implants are often encapsulated in fibrous tissue and show a lack of bone integration which is seen as an essential disadvantage since it can lead to implant subsidence and nonunion [14].

There are new 3D printing production methods such as selective laser melting to fabricate titanium implants whose porous structure comes close to the structure of cancellous bone and whose micro- and even nanostructure promotes cellular migration and bone formation [15]. The EIT cellular titanium cage (EIT Emerging Implant Technologies GmbH, Eisenbahnstraße 84 | 78573 Wurmlingen, Germany) is the latest generation of such a “biologically active” cage design with osteoinductive and osteoconductive properties [6, 13, 15–17]. Even more, its porosity of 80% significantly reduces the modulus of elasticity of titanium to come closer to the elasticity modulus of cancellous bone [18].

There are a limited number of comparative studies between porous titanium and PEEK cages available in literature [6, 16, 19, 20].

In a prospective case series, the radiological and clinical outcomes of this innovative cage for interbody fusion in the cervical spine were evaluated.

## 2. Material and Methods

### 2.1. Study Design

This prospective single-center radiological and clinical study investigates the outcome after one-level anterior cervical discectomy and fusion (ACDF) with an innovative 3D printed titanium cage. Included were patients with cervical disc herniation, cervical stenosis, osteochondrosis, mild spondylolisthesis, mild segmental kyphosis, and spondylodiscitis in whom monosegmental ACDF was indicated.

Surgery was performed by two experienced surgeons. The cages were implanted “stand-alone” and filled with autologous bone marrow harvested from the adjacent vertebral bodies through the holes for the retainer screws. Radiological and clinical data were collected before surgery and 3 and 12 months after surgery.

Patients presenting manifest osteoporosis with a *T*-score below -2 in dual-energy X-ray absorptiometry (DXA) findings and significant instability of the cervical spine leading to a need of additional plating and/or dorsal approach and patients suffering from a tumor were excluded (Table 1).

This clinical trial was approved by the institutional review board. The postoperative follow-up was in accordance with the defined standard for cervical surgical procedures.

### 2.2. Surgical Procedure

All surgeries were performed under general anesthesia and oral (C5-Th1) or nasal (C3-5) intubation in a supine position of the patient on a radiolucent operation table [2]. We performed a common right-sided anterolateral approach with a minimally invasive 2.5 cm incision [2]. Before placing the retainer screws, bone marrow (2 ml) was aspirated from the screw holes. Anterior discectomy was performed with the help of a surgical microscope from this point on in all cases. The disc was removed, and the posterior longitudinal ligament was opened for complete decompression of the dura and the nerve roots. The endplates were carefully detached to expose subchondral bone. After identifying the right cage size, the selected 3D printed titanium cage was filled with the previously aspirated bone marrow and implanted press-fit in the intervertebral disc space under slight distraction of the motion segment [2].

### 2.3. Radiological Assessment

To assess the radiological outcome after surgery, three parameters were considered: RoM, fusion rates, and subsidence.

X-rays of the c-spine were taken in 4 plains (anterior-posterior and lateral view with flexion-extension radiographs) preoperatively and 3 and 12 months postoperatively.

Currently, there is no gold standard for postoperative measurement of fusion after ACDF [21], but it is known that functional radiographs show more precise and reliable measurements than computed tomographies with less inter- and intraobserver variability [22]. The present evaluation was conducted with the FXA™ software (Functional X-ray Analysis, ACES GmbH, Esslingen, Germany). It was developed to precisely determine the RoM of two vertebrae in the human spine [23]. The software calculates RoM values with an accuracy of −0.01° ± 0.03°, which was independently validated and already successfully applied in various international studies [23].

Fusion was defined according to international standards as a RoM < 4° at the operated level on flexion-extension films [24, 25].

### 2.4. Clinical Assessment

Patient-reported outcome measure questionnaires were distributed to the patients before surgery and at both follow-up time points. It contained the validated German version of the NDI [26], the VAS for arm pain and neck pain [27], and an assessment of subjective patient satisfaction (options: “very satisfied,” “satisfied,” and “dissatisfied”).

### 2.5. Statistical Analysis

Significance level was set at a *p* value ≤ 0.05. The data were tested for normal distribution using the Shapiro-Wilk test. We used descriptive statistics for analyses. Statistical differences between time points were tested with the Wilcoxon signed rank test for independent samples. We tested statistical differences between groups using the Mann–Whitney *U* test.

All statistical analyses were performed with IBM SPSS Version 23 (IBM Corp., Armonk, N.Y.).

## 3. Results

A total of 20 patients were eligible for the trial. Two patients received additional plating which led to protocol violation. Of the 18 included patients, 50% were female. Mean age on the day of the operation was 52.2 years. The majority of operated levels were C5/6 (72.2%), followed by C4/5 (16.7%) and C6/7 (11.1%) (Table 2).

### 3.1. Radiological Outcome (RoM, Fusion Rates, and Subsidence)

**Figure 1:**
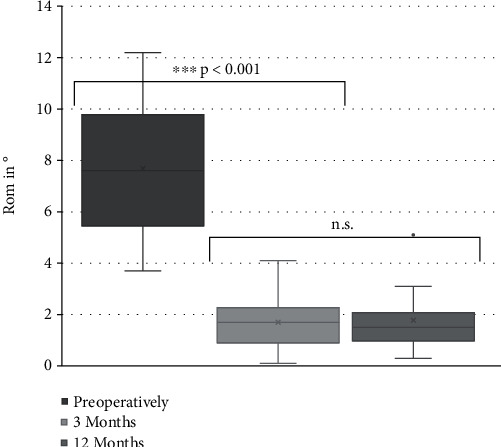
Range of motion. The range of motion preoperatively and at 3 and 12 months postoperatively after implantation of a 3D printed cellular titanium cage. Segmental fusion was achieved 3 months postoperatively. RoM: range of motion; ^∗∗∗^*p* < 0.001; n.s.: not significant.

At baseline, the patients showed a mean RoM of 7.7° ± 2.6° (3.7°–12.2°), which decreased to 1.7° ± 1.1° (0.1°–4.1°) at 3 months postoperatively and 1.8° ± 1.2° (0.3°–5.1°) at 12 months. Between the first two time points and also between the first and last time point, a statistically highly significant difference was seen (both *p* < 0.001). There was no statistically significant difference between the follow-up time points after surgery (*p* = 0.602) (Figure 1).

The fusion rate, defined as <4° RoM at the index level, was 94.4% (17/18 patients) after 3 and also after 12 months.

The remaining patient (Table 2: Pat. #6) had a RoM of 5.9° preoperatively and 4.1° at 3 and 5.1° at 12 months, respectively. By definition, this patient had to be classified as having pseudoarthrosis at 12 months postoperatively; however, the clinical result was satisfactory for the patient.

Mean subsidence was at 0.9 mm ± 0.5 mm (0.1 mm–1.5 mm) 3 months after surgery and at 1.1 mm ± 0.5 mm (0.2 mm–1.8 mm) 12 months after surgery (*p* < 0.001).

### 3.2. Clinical Outcome (NDI/VAS/Subjective Patient Satisfaction)

**Figure 2:**
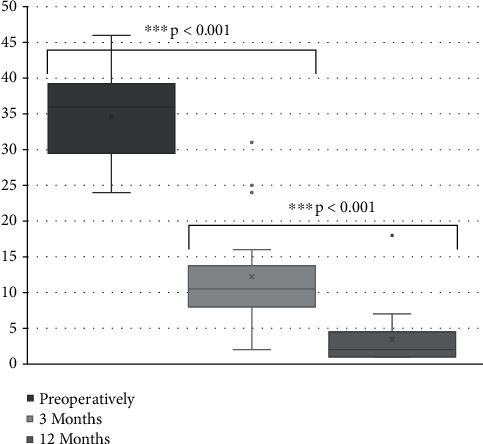
Neck disability index. Neck disability index scores preoperatively and at 3 and 12 months postoperatively. ^∗∗∗^*p* < 0.001.

Baseline mean NDI was 34.6 ± 6.2 (24–46) and decreased to 12.24 ± 7.7 (2–31) 3 months and 3.4 ± 4.1 (1–18) 12 months postoperatively. The differences between all 3 time points were statistically highly significant (*p* < 0.001) as demonstrated in Figure 2.

**Figure 3:**
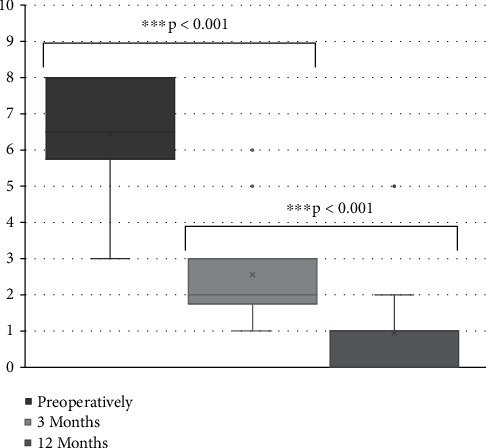
Visual analogue scale for brachialgia. Visual analogue scale scores for brachialgia preoperatively and at 3 and 12 months postoperatively. ^∗∗∗^*p* < 0.001.

The mean VAS for brachialgia improved significantly from 6.4 ± 1.5 (3–8) to 2.6 ± 1.4 (1–6) 3 months after surgery and to 0.9 ± 1.6 (0–5) 12 months after surgery (*p* < 0.001) as shown in Figure 3.

**Figure 4:**
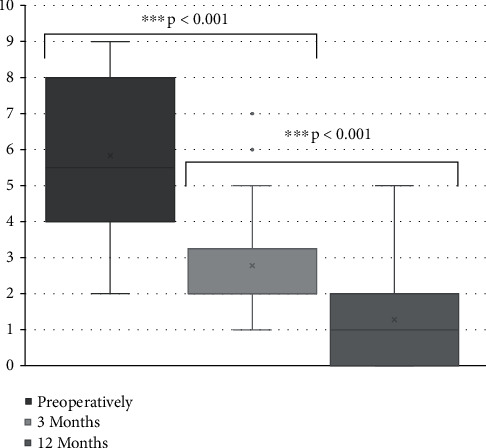
Visual analogue scale for cervicalgia. Visual analogue scale scores for cervicalgia preoperatively and at 3 and 12 months postoperatively. ^∗∗∗^*p* < 0.001.

Mean VAS for cervicalgia started at 5.8 ± 2.2 (2–9) decreasing to 2.8 ± 1.8 (1–7) at 3 months and 1.3 ± 1.4 (0–5) at 12 months postoperatively (*p* < 0.001) (Figure 4).

**Figure 5:**
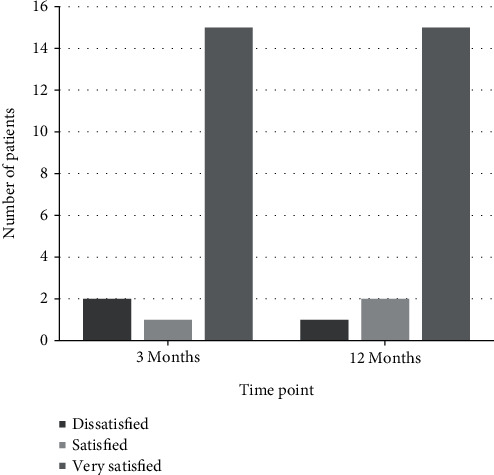
Subjective patient satisfaction. Subjective patient satisfaction at 3 and 12 months postoperatively.

94.4% (17/18) of the patients were satisfied or very satisfied with the clinical outcome after 12 months (Figure 5).

One patient was not satisfied with the result because of persisting pain. However, the radiological result was good with fused segment and remaining RoM of 1.5° after 12 months.

## 4. Discussion

The use of cervical interbody cages has become the gold standard to achieve segmental fusion after discectomy and decompression. In Europe, stand-alone fusion with cages without additional plate fixation is the most commonly used technique [16, 28–30]. Due to several advantages (e.g., cost-efficiency and radiolucency), PEEK-cages are most frequently used internationally.

Usually, these cages have a trapezoid footprint and a ring-type structure to leave space for bone or bone substitutes which are filled in to promote bony fusion of the segment. This either implies the harvesting of autologous bone with all its disadvantages [31] or the use of bone substitutes which cause higher costs [32].

The fusion rates of stand-alone PEEK cages range between 69 and 90% after 6 months or longer [24, 33–35]. However, there is a great variability in the fusion rates as well as in the time span required for a complete and stable fusion [6].

One reason lies in the material properties of PEEK, which does not allow bone ongrowth and which is known to produce a proinflammatory surrounding responsible for apoptosis and cell necrosis [14].

3D printed titanium structures on the other hand have promising material and surface properties which predispose its use for cervical interbody fusion.

The macrostructure can be adjusted to mimic the pore size of cancellous bone on the one hand but providing good primary stability on the other hand. Experimental studies have shown that the surface structure of cellular titanium induces elevated levels of alkaline phosphatase (AP) and osteocalcin production in combination with mesenchymal stem cells in vitro [14]. AP and osteocalcin are known markers of bone formation. Moreover, an increased production of bone morphogenic protein-2 (BMP), BMP-4, BMP-7, and vascular endothelial growth factor (VEGF) which promote the differentiation of mesenchymal stem cells to osteoblasts could be shown in in vitro experiments [14, 36, 37].

Since the cage used in this study is 80% porous, it behaves hydrophilic and is able to absorb and keep fluids. Thus, it predisposes the use of autologous bone marrow as an “endogenous BMP provider.”

The goals of the current study were to analyze the postoperative fusion rate as well as the clinical results after monosegmental stand-alone cage implantations with the use of autologous bone marrow instead of bone substitutes or autologous cancellous bone.

One of the weaknesses of this study is the limited number of patients (*n* = 18) included and the lack of a control group. However, solid fusion according to international standards [22] was achieved in 17/18 patients (94.4%) after 3 and 12 months.

Comparable fusion rates have been published recently by Arts et al. [24]. However, the most striking finding in our study is the fact that the fusion rate after 3 months was the “final” fusion rate, i.e., fusion has been achieved very rapidly in 17/18 patients.

This 3-month fusion rate (which persists also in the 12-month follow-up) compares well to long-term fusion rates published in several international studies with different cage materials and structures and the additional use of autologous bone or bone substitutes [33, 35, 38, 39].

In several prospective studies comparing the fusion rates of porous silicone nitrite or 3D printed titanium cages vs. PEEK cages, interestingly, the fusion rates for PEEK cages were only 66% after 3 months and 90% after 12 months [24, 40, 41].

Even though there is no scientific proof, the combination of a 3D printed titanium cage with autologous bone marrow seems to lead to an accelerated fusion.

Subsidence is one of the problems associated with titanium cage implantation. The reasons for subsidence are mainly poor bone quality, undersizing or oversizing of the implanted cage, and placement of the cage in biomechanically weak areas of the vertebral footprint [34].

There is always a physiological subsidence due to the weight of the head and muscular tension after the patient has returned to a vertical position. Thus, subsidence of up to 3 mm has been defined as being “physiologic” whereas a subsidence of more than 3 mm is considered to be “pathologic” [34].

In a recently published meta-analysis of 71 studies with a total of 4784 patients, the average subsidence rate was 21% [34] and has reached up to 35% in another study according to these definitions [41]. The subsidence in our study was between 0.2 and 1.7 mm after 3 months and 0.2 and 1.8 mm after 12 months. This means that none of our patients showed a pathological subsidence.

It has to be taken into consideration however that we had a highly standardized patient group with a small spectrum of diagnosis and with definitely excluded osteoporosis.

Whether the cage design and properties and/or the early high fusion rate after 3 months are responsible for our low subsidence rate remains open for further studies.

Our clinical results were evaluated with the use of NDI, VAS, and subjective patient satisfaction.

The average baseline NDI dropped from 34.6 to 12.2 and 3.4 after 3 and 12 months, respectively, which was an improvement of 31.5 points after 12 months (*p* < 0.001). This compares well with data published in various recent studies. In comparable studies, the final NDI values were 17 and 16.5, respectively [16, 40].

The pain levels were evaluated separately with the VAS for arm and neck pain.

The improvement of arm pain after 12 months was 5.5 points (from 6.4 to 0.9) and for neck pain 4.5 points (from 5.8 to 1.3) in our study. This also corresponds well with the scientific literature published hitherto [16, 42–44].

Recently, VAS values for arm and neck pain have been defined to represent the “minimum important clinical difference” (MICD) [27, 44–47]. MICD means the minimum improvement of pain level which is subjectively relevant for the patient. In these studies, the MICD range for arm pain was 2.5–4.1 and for neck pain 2.1–2.6 [44]. Thus, an improvement of >4 for arm pain and >2 for neck pain are considered as being superior to the MICD [42, 44].

Our VAS improvements for arm pain (5.5) and for neck pain (4.5) are clearly beyond these thresholds.

These values also correspond to the subjective evaluation of the clinical result reported by the patients. 17/18 patients were satisfied or very satisfied with the outcome.

The one patient who was not satisfied still had a good radiological result.

## 5. Conclusion

This is a prospective case series with a limited number of patients without a control group to evaluate the clinical and radiological results of ACDF with a new 3D printed cellular titanium cage filled with autologous bone marrow. Despite this methodological weakness, we believe that this well-selected patient group operated by 2 surgeons with years of experience in ACDF allows the conclusion that we were able to show that the clinical results are noninferior and, in some aspects, better compared to a series with comparable patient groups published hitherto.

The radiological results suggest a fast and reliable fusion rate which obviously occurs already within the first 3 months postoperatively. This is the most striking finding of this study. One of the weaknesses of this study is the low number of patients, and therefore, the factors responsible for such rapid fusion (cage material and structure, the use of autologous bone marrow, etc.) should be investigated in future studies.

## Figures and Tables

**Table 1:** Inclusion and exclusion criteria.

Inclusion criteria
(i) Patient age > 18 years
(ii) One-level ACDF with aspirated bone marrow from the screw holes
(iii) Minimum follow-up 12 months
(iv) Cervical disc herniation
(v) Cervical stenosis
(vi) Osteochondrosis
(vii) Mild spondylolisthesis and segmental kyphosis
(viii) Spondylodiscitis
Exclusion criteria
(i) Manifest osteoporosis (*T*‐score > −2 in dual-energy X-ray absorptiometry (DXA) findings)
(ii) Significant instability with the need of additional plating and/or dorsal approach
(ii) Two or more levels operated
(iv) Tumor

**Table 2:** Patient demographics.

Pat	Sex	Age	Diagnosis	Segment	RoM pre-OP	NDI pre-OP	VAS-arm pre-OP	VAS-neck pre-OP
1	w	55	Disc herniation	C5/6	8.7	36	8	3
2	w	55	Osteochondrosis	C5/6	7.5	41	7	5
3	m	54	Osteochondrosis/spinal stenosis	C5/6	5.3	24	3	6
4	m	58	Spinal stenosis/myelopathy	C4/5	4.4	26	6	2
5	w	68	Osteochondrosis	C5/6	6.1	38	8	7
6	m	41	Disc herniation	C4/5	5.9	31	6	3
7	w	65	Disc herniation	C5/6	10.9	30	4	6
8	w	59	Disc herniation	C5/6	10.7	39	6	8
9	m	39	Disc herniation	C5/6	8.7	36	8	4
10	m	38	Osteochondrosis/spinal stenosis	C5/6	9.1	40	7	5
11	w	45	Disc herniation/spondylodiscitis	C5/6	4.7	44	6	9
12	m	43	Disc herniation/osteochondrosis	C5/6	10	28	5	8
13	m	47	Spinal stenosis/myelopathy	C5/6	5.1	36	6	9
14	w	55	Disc herniation	C6/7	12.2	31	5	7
15	w	51	Anterolisthesis	C4/5	11.3	46	8	9
16	w	47	Osteochondrosis/spinal stenosis	C5/6	6.3	28	8	5
17	m	60	Osteochondrosis/spinal stenosis	C5/6	3.7	32	7	5
18	m	48	Spinal stenosis	C6/7	7.7	37	8	4

## Data Availability

The data that support the findings of this study are available from the corresponding author upon reasonable request.
